# The Good Waldensian

**Published:** 1872-03

**Authors:** 


### THE GOOD WALDENSIAN.

MANY a heart among the best men and women in America was saddened last year at the intelligence of the death of that good man, the Rev. Dr. Revel, of Florence, Italy. It was so recently that he had been met in New York, so lately heard addressing the crowded "Association Hall," on the great subject of the Church's best interest, that it seemed almost impossible that those lips should so soon be closed in death, which for a long life had been so eloquent in teaching, and urging, and defending the doctrines of the cross. He labored hard, and lovingly, and long, in the great cause of Christian advancement; his was that patient, steady labor, which had in it a tenacity of purpose, which no obstacle could break down, no danger daunt; and yet he had such a loving, gentle heart, so full of all human sympathies, that it was only to know the man, to love him. He died in a good old age, in the harness, an earnest worker to the last, leaving the care of his faithful co-working wife to the lovers of that cause to the promotion of which he had cheerfully given the labors of a lifetime.

With great good taste, and with a generosity well befitting the cause, some New York gentlemen have proposed to make up the sum of five thousand dollars, as a memorial present to the widow, who, without a husband, without children, is left in her old age to make the remnant of life's pilgrimage alone; but how much more cheerily will it be made, when she can all along feel that sustaining encouragement which such a memorial would naturally inspire. The Rev. Doctor Prime, of the "New York Observer," as usual, has a hand in this good movement, and we may be sure he will carry it through. It is an honor and a privilege for all the good to coöperate with him for the short time that the privilege is open.

It is a strong proof of the advancing generosity of this age, that men who have consecrated their life's labor to the service of the Church or the State, all neglectful of their own pecuniary interests, not having sold their opportunities for money, have been liberally cared for in the direction of memorials for their families, who no longer can lean upon them for care and support. Ex-Secretary Stanton worked as no other man worked for what he considered the cause of his country, of liberty, and humanity. He left his family unprovided for; his friends raised his widow a hundred thousand dollars. Another member of a President's cabinet died poor, and promptly a large amount of money was invested for his widow. And never was thirty thousand dollars more promptly, more willingly, nay, gladly and worthily given, than to the widow of one of the best men of the age, the late pastor of a Fifth Avenue Presbyterian Church. These statements are purposely made, to impress upon the minds of all young men that entire consecration to a good cause, to the Church, will bring them adequate rewards here and hereafter, for themselves and the dear ones they may leave behind them; and that therefore they should be stimulated to such entire consecration to the Master's service, in denouncing sin, and vice, and crime, which always lead to disease and premature death, and in the advocacy of virtue and morality, and of that religion which has the promise of the life that now is, and of that which is to come. For after all, the best promotive of longevity, of a healthy life, is the practice of those precepts which the Bible teaches, to wit: economy, industry, and temperance; for "If any would not work, neither shall he eat," and the chief of the Apostles urges to "Be temperate in all things."

